# Host genetics and tumour metastasis

**DOI:** 10.1038/sj.bjc.6601590

**Published:** 2004-02-17

**Authors:** K W Hunter

**Affiliations:** 1 Laboratory of Population Genetics, National Cancer Institute, National Institutes of Health, Building 41, Room D702, 41 Library Drive, Bethesda, MD 20892-5060, USA

**Keywords:** metastasis, cancer, progression, genetics, microarray, gene expression, inefficiency, mouse models, modifiers, quantitative traits, genetic background

## Abstract

Metastasis, the spread and growth of tumours at secondary sites, is an extremely important clinical event, since a majority of cancer mortality is associated with the metastatic tumours, rather than the primary tumour. In spite of the importance of metastasis in the clinical setting, the actual process is extremely inefficient. Millions of tumour cells can be shed into the vasculature daily; yet, few secondary tumours are formed. The classical hypothesis explaining the inefficiency was a series of secondary events occurring in the tumour, resulting in a small subpopulation of cells capable of completing all of the steps required to successfully colonise a distant site. However, recent discoveries demonstrating the ability to predict metastatic propensity from gene expression profiles in bulk tumour tissue are not consistent with only a small subpopulation of cells in the primary tumour acquiring metastatic ability, suggesting that metastatic ability might be pre-programmed in tumours by the initiating oncogenic mutations. Data supporting both of these seemingly incompatible theories exist. Therefore, to reconcile the observed results, additional variables need to be added to the model of metastatic inefficiency. One possible variable that might explain the discrepancies is genetic background effects. Studies have demonstrated that the genetic background on which a tumour arises on can have significant affects on the ability of the tumour to metastasise and on gene expression profiles. Thus, the observations could be reconciled by combining the theories, with genetic background influencing both metastatic efficiency and predictive gene expression profiles, upon which, subsequently, metastasis-promoting mutational and epigenetic events occur. If the genetic background is an important determinant of metastatic efficiency, it would have significant implications for the clinical prediction and treatment of metastatic disease, as well as for the design of potential prevention strategies.

Metastasis is an extraordinarily complex process. To successfully colonise a secondary site, a cancer cell must complete a sequential series of steps before it becomes a clinically detectable lesion. These steps include separation from the primary tumour, invasion through surrounding tissues and basement membranes, entry and survival in the circulation, lymphatics or peritoneal space, arresting in a distant target organ, usually, but not always (Al-Mehdi *et al*, 2000) followed by extravasation into the surrounding tissue, survival in the foreign microenvironment, proliferation, and induction of angiogenesis, all the while evading apoptotic death or immunological response (reviewed in Liotta and Stetler-Stevenson, 1993).

This process is of great importance to the clinical management of cancer, since the majority of cancer mortality is associated with metastatic disease rather than the primary tumour (Liotta and Stetler-Stevenson, 1993). In most cases, cancer patients with localised tumours have significantly better prognoses than those with disseminated tumours. Since recent evidence suggests that the first stages of metastasis can be an early event (Schmidt-Kittler *et al*, 2003) and that 60–70% of patients have initiated the metastatic process by the time of diagnosis, better understanding of the factors leading to tumour dissemination is of vital importance. However, even patients who have no evidence of tumour dissemination at primary diagnosis are at risk for metastatic disease. Approximately one-third of women who are sentinel lymph node negative at the time of surgical resection of the primary breast tumour will subsequently develop clinically detectable secondary tumours (Heimann *et al*, 2000). Even patients with small primary tumours and node-negative status (T1N0) at surgery have a significant (15–25%) chance of developing distant metastases (Heimann and Hellman, 2000).

In spite of the prevalence of secondary tumours in cancer patients, the metastatic process is an extremely inefficient process. To successfully colonise a distant site, a cancer cell must complete all of the steps of the cascade. Failure to complete any step results in the failure to colonise and proliferate. As a result, tumours can shed millions of cells into the bloodstream daily (Butler and Gullino, 1975); yet, very few clinically relevant metastases are formed (Tarin *et al*, 1984). Although many steps in the metastatic process are thought to contribute to metastatic inefficiency, our incomplete understanding of this process suggests that we are aware of some but not all of these key regulatory points. For instance, killing of intravasated cells by haemodynamic forces and sheering has been thought be a major source of metastatic inefficiency (Weiss *et al*, 1992). However, recent evidence suggests that the destruction of tumour cells by haemodynamic force in the vasculature may not always be a major source of metastatic inefficiency. Cells in the bloodstream have been shown to arrest in capillary beds and extravasate with high efficiency and reside dormant in the secondary sites for long periods of time (Luzzi *et al*, 1998), sometimes for years (Riethmuller and Klein, 2001). Micrometastases may form, but the bulk of these preclinical lesions appear to regress (Luzzi *et al*, 1998), probably due to apoptosis (Wong *et al*, 2001).

## GENETIC MODULATION OF METASTASIS

The first suggestion of the role of genetic background as a critical determinant of metastatic potential was derived from transfection experiments. Introduction of proto-oncogenes can induce tumorigenicity and metastatic potential when transfected into NIH-3T3 cells. However, when the same oncogenes were transfected into cell lines derived from different strains of mice, metastatic potential, but not tumorigenicity, was lost (Muschel *et al*, 1985; Tuck *et al*, 1990). These results suggested either that secondary mutations in metastasis-promoting or -suppressing genes were differentially present among the cell lines, or that allelic differences derived from the inbred strain progenitor were capable of modulating metastatic potential.

More compelling evidence for the existence of allelic variation influencing metastatic efficiency comes from experiments from our laboratory. These studies are based on the use of highly metastatic mouse mammary model, the FVB/N-TgN(MMTV-PyVT)^634Mul^ mouse (Guy *et al*, 1992). This animal carries the mouse polyoma virus middle T antigen expressed from the mouse mammary tumour virus enhancer and promoter. Expression of the transgene induces synchronous multi-focal mammary tumours in all of the mammary glands of virgin female animals, and greater than 85% of these animals develop pulmonary metastases by 100 days of age (Guy *et al*, 1992).

To determine whether there was genetic modulation of metastatic progression, the genetic background that the tumour arose on was varied by a simple breeding strategy. The PyVT mouse was bred to a variety of different inbred strains selected from different branches of the mouse phylogenic tree (Beck *et al*, 2000) to survey a broad range of the allelic diversity captured in the inbred strains. The F_1_ progeny were aged to permit tumour induction and potential metastatic dissemination. Subsequently, the lungs were examined to determine whether introduction of allelic variation had an affect on the density of pulmonary metastases, and a wide variation in metastatic efficiency was observed (Lifsted *et al*, 1998). Since all of the tumours were induced by the same genetic event, expression of PyVT, the most likely explanation for this variation is that subtle genetic differences between the strains are affecting the metastasis process.

Further evidence of the effect of background on metastatic efficiency was obtained by genetic mapping experiments. Using quantitative trait mapping strategies, three backcross mapping experiments and a recombinant inbred cross were analysed to identify chromosomal regions associated with metastatic efficiency. Two statistically significant associations were observed, one on chromosome 6 and the other on 19 (Hunter *et al*, 2001). In addition, suggestive associations were reproducibly observed for several other chromosomal regions. The ability to map metastasis efficiency loci within an inbred strain genome argues against random somatic mutations being the major determinant of metastatic efficiency, since each individual animals would retain different sets of alterations, precluding meiotic mapping.

Understanding the events and factors that influence tumour dissemination is clearly of great importance for the development of more effective prevention or clinical interventions. Recent studies have sparked considerable debate in the literature on the subject. Several studies were published that demonstrated the ability to classify primary tumours as metastatic or nonmetastatic, based on gene expression from bulk tumour tissue (van 't Veer *et al*, 2002; Ramaswamy *et al*, 2003). Since a substantial portion of the tumour must exhibit a particular expression pattern to be detectable in microarray experiments, the authors interpret their data to suggest that metastatic capacity is likely to be encoded early in tumorigenesis by the particular collections of oncogenic events that initiate the tumour. As supporting evidence, the authors cite the clinical phenomenon of patients with metastatic disease, but unknown primary cancer (UPC). These patients, estimated at approximately 5% of cases, present with disseminated disease, but have no clinically detectable primary tumour or only a small well-differentiated lesion found at autopsy (Riethmuller and Klein, 2001). The lack of large primary tumour mass could suggest that there were insufficient numbers of cells to achieve the necessary sequence of events predicted by the stochastically driven progression model.

In contrast, the generally accepted progression model predicts that only a small subpopulation of the tumour would attain metastatic capacity and therefore would not be less likely to dominate the average gene expression profile of bulk tumour tissue. However, compelling evidence for the progression model exists. For example, consistent reproducible chromosomal aberrations are often specifically associated with disseminated tumours rather than the primary tumours. The rapidly growing collection of metastasis suppressors, those genes whose reintroduction into tumour cells specifically interferes with metastatic colonisation without affecting primary tumour initiation or growth kinetics, impact virtually every known step in the metastatic process (Kauffman *et al*, 2003; Shevde and Welch, 2003; Steeg, 2003). The statistical likelihood of stochastic events predicted by the model resulting in the appropriate combination of metastasis-associated genomic alterations is small, consistent with the poor efficiency of the process. Although recent evidence suggests that some of these aberrations may occur subsequent to dissemination (Schmidt-Kittler *et al*, 2003), the fact that metastases are often clonal in nature (Fidler and Kripke, 1977) supports the hypothesis that there is a specific subpopulation within the heterogenous primary tumour that these cells originate from.

The truth is likely to be a blend of the models, with additional variables added in. One of these variables is likely the affect of genetic background as a determinant of metastasis. As previously mentioned, we demonstrated that the genetic background on which a cancer arises has a significant affect on the ability of mammary tumours to successfully colonise the lung. In addition, we and others (Eaves *et al*, 2002; Qiu *et al*, 2003) have demonstrated that genetic background significantly influences gene expression, including the metastasis signature genes. The expression of the 17-gene metastasis signature set described by Ramaswamy *et al* (2003) was examined between the high metastatic FVB/NJ background with the low metastatic (NZB/B1NJ × FVB/NJ)F_1_ background. Of the 17 mouse orthologs, 16 were expressed in the PyVT tumour model used in our laboratory. Out of 16, 15 showed the same direction of expression as observed in the human primary *vs* metastasis (Hunter *et al*, 2003; Qiu *et al*, 2003; Ramaswamy *et al*, 2003). Similar results are observed comparing FVB/NJ tumours with another low metastatic genotype ((DBA/2J × FVB/NJ)F_1_; K Hunter, unpublished results). These observations suggest that the propensity of a tumour to metastasise, and the predictive gene expression profile, is at least in part set by the combination of subtle changes in gene function, mediated by polymorphisms in coding sequence, splice sites, promoters, and enhancers, before tumour initiation. Subsequently progressive events such as translocations, deletions, etc., occur to produce rare cells that are capable of completing the metastatic process. The allelic background of the tumour would also likely influence what specific secondary events would be necessary in each individual host genotype to successfully complete the metastatic cascade.

Importantly, the genetic efficiency determinant not only exerts its affects within the tumour cell itself, but also in the primary tumour stroma as well as the microenvironment at distant sites. Target organ microenvironment is known to play an important role in metastasis formation (Fidler, 2002). Tumour cells are known to require normal stroma for important signalling events (Alessandro and Kohn, 2002). Expression of important metastasis-related genes has been shown to be expressed not only in the tumour cells but also in the target tissue (Muller *et al*, 2001). As a result, polymorphisms that alter the function of normal tissue functions, for example, promoter polymorphisms altering cytokine levels, missense polymorphisms affecting adhesion molecule function, alterations in signaling cascades, etc., may be as important a barrier to successful metastatic colonisation as alterations occurring within the tumour cell itself. Alternatively, relevant polymorphisms might indirectly affect important genes by altering epigenetic controls. Several metastasis suppressors have been shown to be epigenetically downregulated during dissemination (e.g. Domann *et al*, 2000), rather than inactivated by mutation or deletion. Since it has been shown that endogenous genes can be differentially imprinted in mouse strains (Jiang *et al*, 1998), polymorphisms that affect more global gene expression by modulating DNA methylation of histone modification must also be considered as potential metastasis modulating functions.

The growing evidence suggesting that the majority of tumour cells are capable of extravasating (Naumov *et al*, 2002) suggest that proliferation in the secondary sites may in fact be one of the most important determinants to whether cells proliferate into a secondary tumour or undergo apoptosis. Since the growth of disseminated cells to clinically relevant macroscopic lesions is dependent upon angiogenesis, the effect of genetics on this process might be another important source of metastatic efficiency modulation. Inbred strains of mice are known to be different in their angiogenic response to at least some growth factors (Rohan *et al*, 2000). Differences in the ability of the target stroma in different genotypes to support angiogenic conversion from microscopic to macroscopic secondary lesions in response to tumour-secreted growth factors might therefore play an important role in the efficiency of the development of clinically relevant secondary tumours. Furthermore, it is conceivable that allelic variation may affect escape from immune surveillance. Subtle variations in the ability of the host to mount an effective cytolytic defense, coupled with the ability of highly malignant cells to downregulate tumour-specific antigens (Schirrmacher *et al*, 1982), might also play an important role in metastatic efficiency. It is unclear at present which of these, or other cellular or molecular processes or combinations of all, might be responsible for genetic modulation of metastasis. Clearly, this complex and complicated process will require a great deal of additional research to explore and characterise the critical interplay between inherited, somatic, and epigenetic interactions that influence metastatic progression.

## IMPLICATIONS

These observations, particularly the microarray data, have important implications for metastasis detection and management. If genetic background is a major influence on metastatic potential, as measured by predictive gene expression patterns in normal and tumour tissue, it suggests that, like cancer susceptibility, there may be individuals or families present in the human population that are more susceptible to disseminated disease. It may therefore be possible to identify these individuals before they develop neoplastic disease, so that they might be more aggressively treated with neo-adjuvant therapies immediately upon diagnosis of the primary tumour. Alternatively, since tumour dissemination often appears to be an early event, it is theoretically possible that a chemoprevention regime might be developed that would prevent tumour metastasis before the primary tumour was clinically apparent, enabling the bulk of human cancer to be cured by surgical resection.

In conclusion, the identity of the genomic elements in the host background modifying metastatic efficiency is currently unknown. They clearly warrant further investigations, since the majority of the genetically defined regions are not associated with known metastasis-suppressor genes. The metastasis suppressors that are associated with our genetically defined regions do not have any apparent molecular defects nor expression level differences between the high and low metastatic genotypes (Park *et al*, 2002; Qiu *et al*, 2003). Identification and characterisation of these metastasis efficiency-modifier genes may therefore yield novel targets to develop chemoprevention agents or antimetastatic therapies. Preliminary evidence of the feasibility of such a strategy is currently ongoing in our laboratory. Using a small-molecule agent, we have demonstrated a significant reduction in the efficiency of pulmonary colonisation, as well as modulation of the expression profile of an independent set of metastasis-associated genes (Yang, Lukes, Rouse, Lancaster, and Hunter, manuscript in preparation). The new strategies could be developed to either kill occult metastases or possibly increase the inefficiency of the myriad tasks necessary to generate a clinically relevant metastasis to the point where the odds of solitary, dispersed cancer cells successfully completing the metastatic cascade to become clinically relevant lesions approach zero.
